# Assessment of dental anxiety and dental phobia among adults in Lebanon

**DOI:** 10.1186/s12903-021-01409-2

**Published:** 2021-02-04

**Authors:** Hiba Kassem El Hajj, Youssef Fares, Linda Abou-Abbas

**Affiliations:** Neuroscience Research Center, Faculty of Medical Sciences, Lebanese University, Beirut, Lebanon

**Keywords:** Modified dental anxiety scale, MDAS, Psychometric evaluation, Lebanese, Arabic version

## Abstract

**Background:**

Dental anxiety continues to be a widespread problem affecting adult populations. The primary aim of our study was to evaluate the psychometric properties of the Lebanese Arabic version of the Modified Dental Anxiety Scale (MDAS-A) and to identify the optimal cut-off for assessing dental anxiety and dental phobia among adults in Lebanon. In addition, we sought to assess dental anxiety and phobia as well as their correlates among Lebanese adult patients.

**Methods:**

A cross-sectional study was carried out on a sample of 451 dental adult patients aged between 18 and 65 years old. Information about demographic characteristics, previous bad dental experience, trauma’s experience period, perception of a periodontal problem, sensation of nausea during dental treatment, the MDAS-A scale, and the Visual Analogue Scale for anxiety (VAS-A) were collected.

**Results:**

MDAS-A exhibited evidence of adequate psychometric properties. The optimal cut-off was 12 for dental anxiety and 14 for dental phobia. Out of the total sample, 31.5% suffered from dental anxiety while 22.4% had a dental phobia. Multivariable analysis showed that the odds of dental anxiety and phobia were higher among females compared to males. Also, patients suffering from periodontal problem perceptions, bad dental experiences during childhood and adolescence, and the sensation of nausea during dental treatment were at a higher risk of developing dental anxiety and phobia compared to their counterparts. However, a higher level of education was found to be a protective factor against dental phobia among Lebanese adult patients.

**Conclusion:**

The MDAS-A scale is a suitable tool for the routine assessment of dental anxiety and phobia among Lebanese adult patients. Identifying patients with dental anxiety at the earliest opportunity is of utmost importance for delivering successful dental care.

## Background

Despite the recent innovation and technological advances in modern dentistry, dental anxiety continues to be a widespread problem affecting child and adult populations. Several researchers have described a “dynamic vicious cycle” relating dental anxiety to bad oral health [1–3]. Patients with dental anxiety tend to delay or avoid dental care which will result in worsening their oral health. Progressive worsening of the untreated oral diseases coupled with the feeling of guilt, shame, or inferiority contribute to a further increase in dental anxiety, and the vicious cycle continues [3]. On the other hand, patients with dental anxiety are a considerable source of stress that can compromise the dentists’ clinical performance [4]. Thus, there is a need to identify patients with dental anxiety before treatment initiation. This can help dental care providers to break the vicious cycle and to provide effective treatment [5, 6].

A clinical diagnostic interview is required to establish a definite diagnosis of dental anxiety. Furthermore, having a valid and reliable instrument is of great importance in clinical settings. In response to this need, researchers have developed various specific instruments to evaluate dental anxiety [7, 8]. One of them is the Modified Dental Anxiety Scale (MDAS) which is used frequently for this purpose [9].

MDAS is an adaptation of the original Corah’s dental anxiety scale (CDAS) which is a 4-item tool requesting patients to estimate their anxiety levels in four different dental situations [10]. MDAS was proposed by Humphris et al. to improve the original CDAS by adding a question about getting local anesthetic injections and requesting the possible replies to each question in a Likert scale ranging from “not anxious” to “extremely anxious” [9]. MDAS was initially established in the English language and authors have revealed good psychometric properties among adults in several English-speaking countries such as United Kingdom [9, 11, 12], Ireland [11], and Wales [13].

Due to the influence of linguistic and cultural differences, MDAS has been translated and adapted to several languages including Chinese [14], Nepali [15], Malay [16], Greek [17], Spanish [18], Tamil [19], Turkish [20], Italian [21] and Arabic [5, 22–24]. In the Arab world, the psychometric properties of the MDAS were tested in two groups of adult Arabic-speaking individuals in Saudi Arabia only [5, 24].

Given that the spoken Arabic dialects differ considerably across the Arab cultures and countries, it is recommended to adapt and validate the translated measuring system in other Arabic countries. Additionally, testing another Arabic version can assign more information about its validity and reliability. Thus, the current study aims to test the psychometric properties of the Lebanese Arabic version of the MDAS and to identify the cut-off to determine anxiety and phobia. Besides, we sought to assess dental anxiety and phobia and to determine their associated factors in a group of Lebanese adults.

## Methods

### Study design and population

This was a cross-sectional study conducted from March until June 2019. Patients were recruited at 29 private Lebanese dental clinics. The inclusion criteria consisted of being Lebanese, aged between 18 and 65 years, and able to write and read the Arabic language. Pregnant women as well as patients with malignant diseases and mental disabilities were excluded from the study. The guideline for Strengthening the Reporting of Observational Studies in Epidemiology (STROBE) was followed when reporting this study [25].

### Procedure

Information about the demographic characteristics (age, gender, marital status, education level), previous bad dental experience, trauma’s experience period, perception of periodontal problem, sensation of nausea during dental treatment, the Arabic version of the Modified Dental Anxiety Scale (MDAS-A), and the Visual Analogue Scale (VAS-A) were collected from the participants. To determine the test–retest reliability of the MDAS-A scale, 30 patients were recruited to complete the MDAS-A scale twice. The time between the two tests was 2 weeks. The questionnaires were distributed to 500 patients, 451 were completed which corresponded to an overall effective response rate of 90.2%.

### Study measurements

#### Modified Dental Anxiety Scale (MDAS)

The MDAS consists of 5 questions to measure the degree of dental anxiety in 5 situations: preparing for a dental visit, waiting in the dentist's office for treatment, sitting in the dental chair for drilling, getting ready in the dental chair for scaling, and preparing for local anesthetic injection. Evaluations range from “non-anxious” [1] to “extremely anxious” [5]. The total score ranges from 5 to 25 while higher scores indicating severe anxiety.

Items of the MDAS have been translated and adapted to the Arabic language in Saudi Arabia [5]. The Arabic dialects differ across the Arab countries, so there was a need to cross-culturally adapt the Arabic version to the Lebanese dialect. Thus, a review committee consisting of two dentists and an epidemiologist, who are native English speakers, revised the Arabic version. They were asked to consider whether the items are a good fit for the Lebanese adult population. Only, the fourth item of the MDAS Arabic version has been an area of discussion between the review committee who agreed that the Lebanese population could not understand the Arabic translation of “teeth scaled and polished”. Therefore, this sentence was glossed with an explanation of “cleaning” in brackets after consensus between committee members. Finally, a new Arabic version adapted to the Lebanese culture was produced and referred to as MDAS-A.

A pilot study on a group of thirty patients was conducted to check the clarity of the Arabic items. The test was completed within 4 min and no difficulties were reported.

#### The Visual Analogue Scale for Anxiety (VAS‐A)

To study the validity, patients rated their current level of dental anxiety on a 100 mm scale, where zero indicated “not at all anxious” and 100 indicated “extreme dental anxiety”. Previous studies have confirmed the validity and reliability of VAS-A in assessing dental anxiety [26–28]. Thus, researchers can use the VAS-A to assess dental anxiety, with the cut-off of ≥ 51 for anxiety and ≥ 70 for phobia [28].

### Statistical analysis

The statistical software SPSS version 22.0 was used for analyses. Means and standard deviations (SD) were used to report descriptive statistics for continuous variables and frequency with percentages for categorical variables. Cronbach’s alpha was used to assess the internal consistency of the MDAS-A scale. The test–retest reliability was assessed by calculating the intra-class correlation coefficient (ICC). ICC values between 0.40 and 0.59 are considered fair, values between 0.60 and 0.74 are good and between 0.75 and 1.0 are excellent [29]. Sample size guidance indicated that 200–300 participants per scale item would be adequate for establishing sufficient evidence of scale validity and reliability [30]. Thus, the total group was randomly divided into two groups using the randomization function on SPSS 22.0. In the first random-half sub-sample (n = 225), the exploratory factor analysis (EFA) was performed through the principal components analysis using Varimax rotation. Confirmatory factor analysis (CFA) was performed in the second random-half sub-sample (n = 226) using the Amos software version 22.0. The goodness-of-fit of the models were evaluated using Chi-square (χ^2^) and degrees of freedom (df), Root Mean Square Error of Approximation (RMSEA), Goodness of Fit Index (GFI), and Comparative Fit Index (CFI). Spearman correlation coefficient was used to assess convergent validity by correlating MDAS-A to VAS-A scores in the total sample. MDAS-A total scores were compared between patients with and without anxiety as well as phobic and non-phobic participants using independent-samples *t*-test to determine the criterion validity of the scale. To find the optimal cut-off value for detecting dental anxiety and phobia, the receiver-operating characteristic (ROC) curve was applied and the Youden index was calculated. Multivariable logistic regression analyses were performed to identify associated factors of dental anxiety and phobia. Adjusted odds ratio and their 95% confidence intervals were reported. The final logistic regression model was reached after ensuring the adequacy of our data using the Hosmer and Lemeshow test. All statistical tests were two-sided, and the significance level was set at 0.05.

## Results

### Baseline characteristics of the study sample

Table 1 displays the demographic characteristics of the whole sample and the two split samples. The mean age was 34.13 (SD = 10.96) ranging from 18 to 65 years. Of the total, 65.5% were females and 60.5% had a university level of education. There were no significant differences in age, gender, and education level between the two split samples.

**Table 1:** Characteristics of the whole study group and the Random Split-Half Samples

	All sample (N = 451)	Split Sample 1 (n = 225)	Split Sample 2 (n = 226)	*P* value
Age mean (SD)	34.1 (11.0)	34.7 (11.5)	33.5(10.3)	0.275
Gender n (%)				0.972
Male	158 (35.0)	79 (35.1)	79 (35.0)	
Female	293 (65.0)	146 (64.9)	147 (65.0)	
Educational level n (%)				0.684
Primary	27 (6.0)	12 (5.3)	15 (6.6)	
Complementary	76 (16.9)	41 (18.2)	35 (15.5)	
Secondary	75 (16.6)	34 (15.1)	41 (18.1)	
University	273 (60.5)	138 (61.3)	135 (59.7)	

N or n Frequency, % percentage, SD Standard deviation, *P* value < 0.05 is significant

### Reliability of the MDAS-A scale

The MDAS-A total mean score was 10.1 with a standard deviation of 4.78. The internal consistency of the MDAS-A total scale was calculated using Cronbach's alpha. For the total sample of 451 participants, the MDAS-A demonstrated high internal consistency with an alpha coefficient of 0.91. The Corrected–item to total correlation coefficients ranged from 0.68 to 0.82 indicating that each item contributes significantly to the total MDAS-A scale. Deleting an item from the construct did not significantly change the alpha level (Table 2). The test–retest ICCs were calculated for the five individual items and the total score. The results of the MDAS-A total score were excellent with an ICC of 0.932 suggesting strong reproducibility (Table 3).

**Table 2:** Internal consistency of the MDAS-A scale (N = 451)

MDAS-A item	Mean (SD)	Scale mean if item deleted	Scale variance if item deleted	Corrected item-total correlation	Cronbach's alpha if item deleted	Cronbach's alpha
Visit tomorrow	1.8 (1.01)	8.26	15.28	0.82	0.88	
Waiting room	1.8 (1.04)	8.28	15.14	0.81	0.88	
Use of drill	2.2 (1.17)	7.85	14.17	0.82	0.87	
Scale and polish	1.91 (1.11)	8.15	15.62	0.68	0.90	
Injection	2.34 (1.23)	7.72	14.50	0.72	0.90	
MDAS-A total	10.1 (4.78)					0.91

MDAS-A: Lebanese Arabic version of the Modified Dental Anxiety Scale; SD, Standard deviation

**Table 3:** Intraclass correlation coefficients for test–retest reliability of the five items and total score of the MDAS-A (n = 30)

MDAS-A item	ICC	95% CI
Visit tomorrow	0.938	0.870–0.970
Waiting room	0.929	0.851–0.966
Use of drill	0.780	0.538–0.895
Scale and polish	0.638	0.239–0.828
Injection	0.858	0.702–0.933
MDAS-A total	0.932	0.857–0.968

MDAS-A: Lebanese Arabic version of the Modified Dental Anxiety Scale, ICC Intraclass correlation coefficient, n frequency

### Factor structure of the MDAS-A

The first split sample underwent the exploratory factor analysis of the MDAS-A. The value of the KMO measure was 0.859 which indicated suitable sampling adequacy, and Bartlett’s Test of sphericity was statistically significant. Hence, the data was deemed suitable for factor analysis. A one-factor structure was derived which included all the items of the scale and accounted for 70.55% of total variance with an eigenvalue of 3.53 (Table 4).

**Table 4:** Exploratory factor analysis of the MDAS Scale (n = 225)

MDAS-A item	Communality
Visit tomorrow	0.800
Waiting room	0.765
Use of drill	0.777
Scale and polish	0.574
Injection	0.611
Eigenvalue	3.53
Percentage of explained variance	70.55

n frequency, Extraction method: principal component analysis; Rotation method: Varimax with Kaiser normalization

**Fig. 1:**
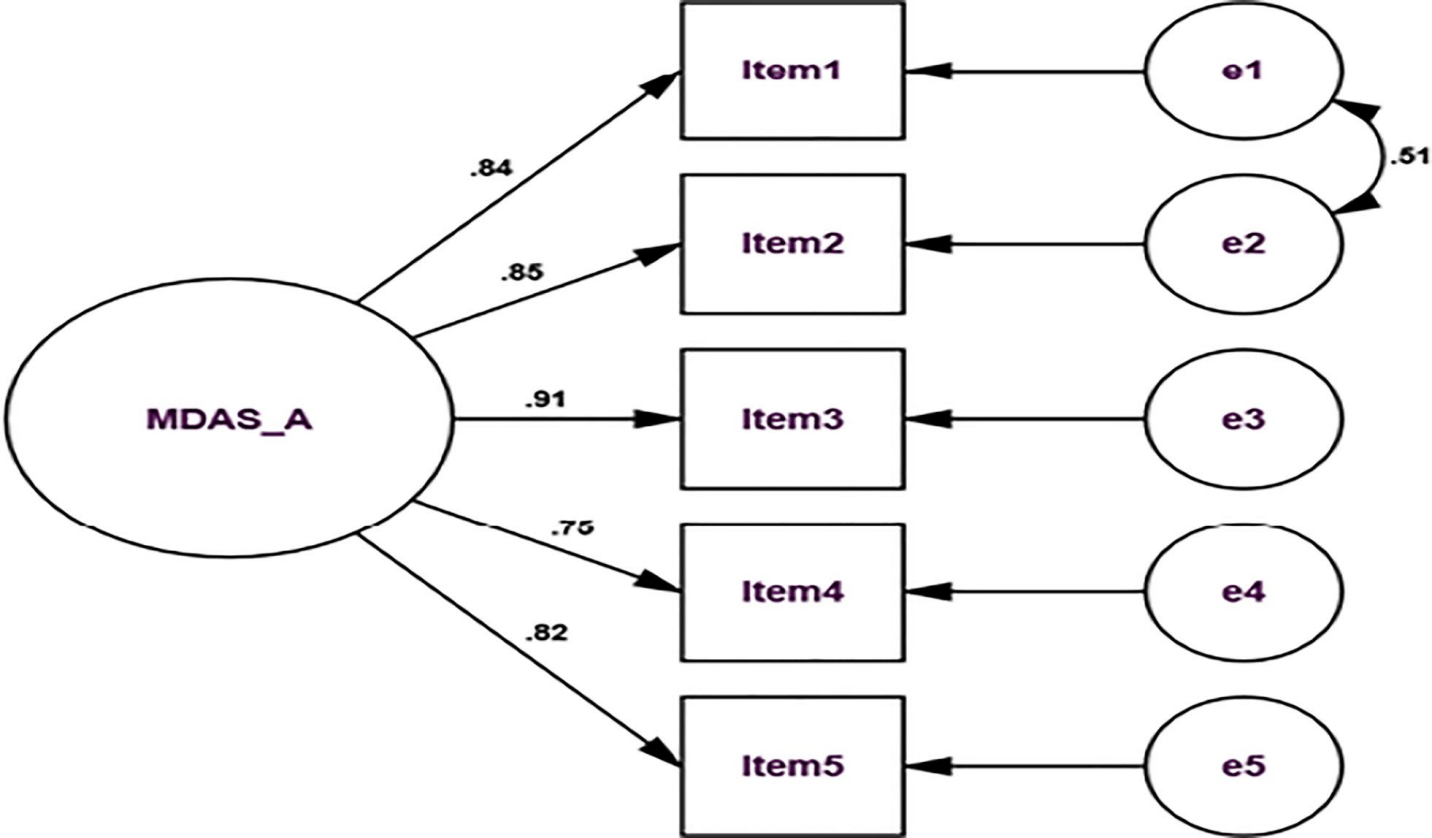
One-factor model of the Arabic version of the Modified Dental Anxiety Scale (MDAS-A)

Confirmatory factor analysis was performed to determine the unidimensional model of the MDAS-A, that is, all items loading onto a single latent variable as suggested by the EFA (Model A). The one-factor model displayed an unsatisfactory fit which was significant. Inspection of the modification indices suggested adding error covariance between items 1 and 2 of the MDAS-A (Model B). This modification resulted in a significant improvement of the fit indices (Table 5). All standardized factor loadings for the one-factor model were significant at *P* < 0.01 suggesting a satisfactory factor loading (Fig. 1).

**Table 5:** Summary statistics of the whole model fit for the unidimensional factor of the MDAS-A

	χ^2^	χ^2^/df	CFI	GFI	RMSEA
Model A	37.97^†^	7.59	0.96	0.931	0.171
Model B*	5.89^‡^	1.47	0.998	0.990	0.046

χ^2^ chi-square, df degree of freedom, RMSEA Root Mean Square Error of Approximation**,** GFI Goodness of Fit Index, CFI Comparative Fit Index, * as Model A with the correlation between the two residual errors for the first two MDAS items, ^†^*P* value ˂0.0001, ^‡^*P* value = 0.207

### Convergent validity of the MDAS-A

Convergent validity of the scale was assessed using Spearman’s correlation coefficient relating the total MDAS-A score and VAS-A score. A statistically significant correlation was found (r = 0.72 with a *P* value < 0.0001) indicating a strong positive correlation and thus, a good convergent validity. Spearman correlations between VAS-A and individual items of the MDAS-A were also significant (*P* value < 0.0001).

### Criterion validity of the MDAS-A

**Fig. 2:**
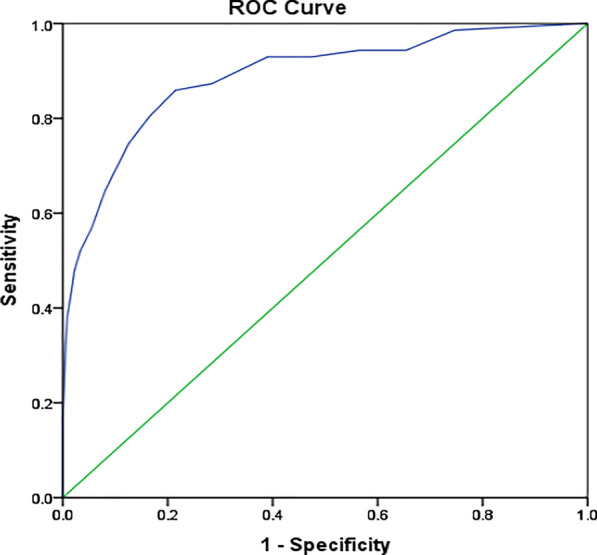
Receiver Operating Characteristic (ROC) Curve revealing Sensitivity as a Function of 1-Specificity of the Modified dental anxiety scale (MDAS) against the visual analogue scale (VAS). The VAS threshold of dental anxiety is 51 [28]

**Fig. 3:**
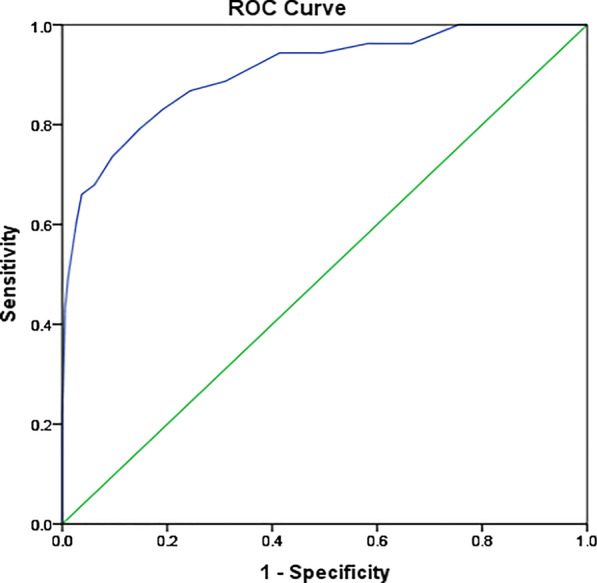
Receiver Operating Characteristic (ROC) Curve revealing Sensitivity as a Function of 1-Specificity of the Modified dental anxiety scale (MDAS) against the visual analogue scale (VAS). The VAS threshold of dental phobia is 70 [28]

Mean scores on the MDAS-A scale were compared between those diagnosed with and without anxiety (made through the VAS-A ≥ 51) using the independent t-test. A statistically significant mean difference was found between the two groups with higher scores for patients with dental anxiety compared to patients without dental anxiety (16.6 vs 8.8, *P* value ˂0.0001). Mean scores on the MDAS-A scale were also compared between those diagnosed with and without phobia (made through the VAS-A ≥ 70) using the independent T-test. A statistically significant mean difference was found between patients with and without anxiety with higher scores for patients with anxiety (16.6 vs 8.8, *P* value ˂0.0001). The same was revealed between patients with and without phobia with higher scores for phobic patients (17.7 vs 9.0, *P* value ˂0.0001). These results indicate that MDAS-A has good discriminant validity. The ROC curve showed a significant area under the curve (AUC) for dental anxiety (AUC = 0.89, 95% CI (confidence interval): 0.84–0.93) (Fig. 2) and dental phobia (AUC = 0.91, 95% CI 0.86–0.95) (Fig. 3). The optimal cut-off score to determine the dental anxiety on the MDAS-A scale as shown by the ROC curve was 12, with a sensitivity of 86% and a specificity of 79%. While for dental phobia, it was 14 with a sensitivity of 79% and a specificity of 85%.

### Assessment of dental anxiety and phobia and their associated factors in the total sample of Lebanese adults

Of the total sample, 31.5% suffered from dental anxiety while 22.4% had a dental phobia. Multivariable analysis showed that the odds of dental anxiety was 2 times higher among females compared to male (adjusted OR 2.05 with 95% CI of 1.23 to 3.40). Patients with a previous bad experience during childhood and adolescence were 3.45 and 2.18 times more likely to suffer from anxiety compared to their counterparts with no previous bad experience (adjusted OR 3.45 with 95% CI of 1.63–7.30 and adjusted OR 2.18 with 95% CI of 1.20–3.94 for childhood and adolescence respectively). Patients with a perceived periodontal problem were 1.57 times more likely to suffer from dental anxiety (adjusted OR 1.57 with 95% CI of 1.18 to 2.10). Finally, the sensation of nausea during dental treatment was found to be a risk factor for dental anxiety (adjusted OR 3.85 with 95% CI of 2.31 to 6.40) (Table 6).

**Table 6:** Factors associated with dental anxiety among Lebanese patients

	All (N = 451)		
	OR adj	95% CI	*P* value
Gender			
Male^†^	1		0.006*
Female	2.05	1.23–3.40	
Trauma’s experience period			
No previous experience^†^	1		0.02*
During childhood	3.45	1.63–7.30	
During adolescence	2.18	1.20–3.94	
During adulthood	1.18	0.61–2.30	
Periodontal problem			
No^†^	1		0.002*
Yes	1.57	1.18–2.10	
Sensation of nausea during dental treatment			
No^†^	1		< 0.0001*
Yes	3.85	2.31–6.40	

OR adj, adjusted odds ratio; CI, confidence interval

^†^Reference group, factors entered into the model: age, gender, marital status, educational level, the trauma’s experience period, the perception of a periodontal problem and the sensation of nausea, *P value < 0.05 is considered statistically significant

Concerning dental phobia, females were at higher risk compared to males (adjusted OR 2.55 with 95% CI of 1.41–4.61). Patients with a previous bad experience during childhood and adolescence were more likely to suffer from dental anxiety compared to their counterparts with no previous bad experience (adjusted OR 3.74 with 95% CI of 1.72–8.11 and adjusted OR 1.94 with 95% CI of 1.01–3.73 for childhood and adolescence respectively). Patients with a perceived periodontal problem were more likely to suffer from dental phobia (adjusted OR 1.38 with 95% CI of 1.01 to 1.88). The sensation of nausea during dental treatment was found to be a risk factor for dental phobia (adjusted OR 3.00 with 95% CI of 1.76 to 5.11). Finally, a higher educational level was found to be a protective factor against dental phobia (adjusted OR 0.76 with 95% CI of 0.58 to 0.98) (Table 7).

**Table 7:** Factors associated with dental phobia among Lebanese patients

	All (N = 451)		
	OR adj	95% CI	*P* value
Gender			0.002*
Male^†^	1		
Female	2.55	1.41–4.61	
Trauma’s experience period			0.006*
No previous experience^†^	1		
During childhood	3.74	1.72–8.11	
During adolescence	1.94	1.01–3.73	
During adulthood	1.45	0.70–2.98	
Perception of a periodontal problem			0.046*
No^†^	1		
Yes	1.38	1.01–1.88	
Sensation of nausea during dental treatment			< 0.0001*
No^†^	1		
Yes	3.00	1.76–5.11	
Education level			0.032
≤ 12 years	1.00		
˃ 12 years	0.76	0.58–0.98	

OR adj, adjusted odds ratio; CI, confidence interval

^†^Reference group, factors entered into the model: age, gender, marital status, educational level, Trauma’s experience period, perception of a periodontal problem, and the sensation of nausea during dental treatment, *P value < 0.05 is considered statistically significant

## Discussion

“Dental anxiety” and “dental phobia” represent a significant challenge for both patients and dentists. These two terms are often used interchangeably; however, they do have distinct differences. Dental anxiety is defined as a patient's specific reaction toward stress associated with dental treatment in which the stimulus is unknown, vague, or not present at the moment [12]. On the other hand, dental phobia is characterized by an extreme and persistent fear of clearly discernible, circumscribed objects or situations in dental setting which results in the individual's avoidance of attending a dentist at all costs, unless possibly when a physical problem becomes overwhelming [31]. Dental practitioners are recommended to assess dental anxiety and dental phobia during clinical assessment using a well structured and psychometrically valid scale that could measure the subjective experience of dental anxiety and phobia in an objective way [31]. In response to this need, the purpose of the present study was to evaluate the psychometric properties of a Lebanese Arabic version of the MDAS and to assess dental anxiety and dental phobia as well as their correlates in a group of Lebanese adults patients. The Lebanese Arabic version of the MDAS exhibited good validity and reliability evidence. Our results also revealed that female patients were at higher risk of developing dental anxiety and dental phobia compared to males. Besides, previous bad experiences during childhood and adolescence, periodontal problem perception, and suffering from a sensation of nausea during dental treatment were risk factors for dental anxiety and dental phobia. However, patients with a higher educational level were found to be at lower risk of developing dental phobia.

Results of our study revealed a good level of internal consistency (Cronbach’s alpha: 0.91) for MDAS-A. This comes in consistency with the study of Humphris in 1995 (Cronbach’s alpha: 0.84 to 0.90) [9]. Moreover, the cross-culturally adapted studies of the MDAS such as the Romanian (0.90) [32], Turkish (0.91) [33], Greek (0.90) [34], United Kingdom (0.917) [13], Italian (0.92) [21], and Japanese versions (0.88) [35] reported similar results. The MDAS-A revealed strong reproducibility over time with an ICC of 0.93. This is consistent with a study that showed a high degree of accordance between test and retest reproducibility (0.81 to 0.82) [9], as well as the study conducted in Japan that reported an ICC of 0.91 [36].

The exploratory factor analysis revealed one factor that accounts for 70.55% of the variance. Most studies that inspected the structural validity of the MDAS through EFA revealed strong evidence for a one-factor structure [5, 16]. The present study adds to the multiple publications on the psychometric properties of the Arabic version of the MDAS by investigating its factorial validity through Structural Equation Modeling (SEM) procedures, suggesting that all items support a one-factor structure. The one-factor structure did not have a good fit norm. Thus, it was inspected by ways of the modification indices which revealed proof of a significant correlation among the two residual errors of the first two questions of the MDAS-A. An inspection of the first 2 items proposes that they have some overlap as they both pay particular attention to the expectation of anxiety before dental treatment. So, computing error covariance between the first two items improved considerably the fit indices. The consistency of our findings with those previously reported suggests that the MDAS had good construct validity.

A strong positive correlation linking MDAS-A and VAS-A was revealed suggesting a good level of convergent validity. Such results were also reported by Appukuttan et al. [19]. Results of different studies revealed moderate correlations between the MDAS and dentists’ observations (0.4 to 0.66) [9, 34, 37]. In our study, the strength of the correlation between the dentist’s observations and MDAS scoring was also moderate (results not shown). This is justified by the reality that some patients try to hide their dental anxiety in order not to interrupt the treatment process or feel ashamed to share their anxieties with their dentists.

Since no Lebanese clinics are specialized in diagnosing patients with dental anxiety or phobia, the VAS-A was used in the assessment of dental patients as suggested by Facco et al. [28]. Hence, ROC curve analysis was allowed to estimate the cut-off values for anxiety and phobia in MDAS‐A that best fitted VAS-A data whereby anxiety and phobia were defined by a score ≥ 51 and a score ≥ 70 on the VAS-A respectively. The ROC curve revealed the discriminant validity of the MDAS-A scale. The AUC value was 0.89 for dental anxiety (95% CI 0.84–0.93) and 0.91 for dental phobia (95% CI 0.86–0.95) indicating that the MDAS-A distinguishes between patients with and without dental anxiety or phobia. The optimal cut-off point to distinguish between patients with and without dental anxiety is 12 with a sensitivity of 86% and a specificity of 79%. While for dental phobia, it was 14 with a sensitivity of 79% and a specificity of 85%. Previous studies have reported various cut off for patients with dental anxiety or phobia with different levels of specificity and sensitivity, maybe due to the use of different diagnostic criteria for the dental anxiety or the populations’ differences in their expressing of dental anxiety. The cut-off point of 19, which has been widely used to spot phobic dental patients [9, 33, 38], has a high specificity (99.5%) but beneath sensitivity (0.43). Besides, the cut-off of 15 which has been recommended in two studies conducted in Saudi Arabia [24] and Turkey [33] produced a high specificity (94%) but lower sensitivity (0.68). Thus, we recommend a cut-off point of 14 to screen for dental phobia in a Lebanese population as it had the finest union of sensitivity and specificity. The MDAS-A should be validated against a gold standard such as the Structured Clinical Interview for DSM-V to draw definitive conclusions.

Our results revealed that 31.5% suffered from dental anxiety while 22.4% had a dental phobia. Studies have reported a wide range of dental anxiety and dental phobia prevalence estimates in adult populations. This might be related to the fact that the prevalence estimates would differ considerably depending upon the cut-off points used to define a case of dental anxiety or dental phobia. It might also be related to the differences in the scales used to assess dental anxiety or dental phobia. Another possible may be related to culture or to the methodological variations in terms of study design or sampling methods across studies [31]. Therefore, our results cannot be compared with others.

The levels of dental anxiety and phobia were higher among females compared to males. This corroborates with the results of previous studies [4, 5, 16, 21, 35, 39, 40]. The perceived gender difference in anxiety and phobia could be attributed to a combination of emotional and social factors. Women are more able to express their feelings of panic, fear of pain, stress, and depression toward dental procedures while men felt embarrassed and tend to hide their anxiety and phobia toward dentistry [41]. Furthermore, past traumatic dental experiences during childhood and adolescence seem to play an important factor in increasing dental anxiety and phobia. This result is consistent with previous studies [4, 16, 21, 35]. In fact, since dental memory is extremely powerful, the upcoming painless dental experience cannot overcome previous bad dental experiences [5]. Thus, the previous unpleasant dental experience can influence the behavioral intention to visit a dentist [42], thereby increasing the patient’s dental anxiety.

We also found that patients with self-perception of periodontal problems were more anxious which was consistent with a study conducted in Germany [40]. However, patients with a higher educational level suffered less from dental phobia when compared to their less-educated counterparts. This is consistent with the studies conducted by Erten et al. [43] and Do Nascimento et al. [44] and could be attributed to greater awareness and better oral health of the patients with a high level of education as well as their regular visit to dental clinics.

### Recommendations for dental routine practice

Since dental anxiety is a real worldwide problem that results in avoidance of dental care and treatment, it is of great importance to use a valid and reliable instrument that could help in identifying dental anxiety and phobia. The use of the MDAS-A could help health care providers in detecting patients with dental anxiety or phobia and to initiate effective strategies to combat anxiety and phobia among adult patients seeking dental care.

The results of the present study need to be considered in light of several methodological limitations. The possibility of selection bias due to the convenience sampling procedure used to select patients and the absence of randomization. We should emphasize that this translated Arabic-language form cannot be appropriate to different Arabic-speaking societies. Other attempts should be considered to adjust the scale to the linguistic characteristics of other Arabic-speaking communities.

## Conclusion

This study was the first to explore the psychometric properties of the Arabic version of the MDAS in Lebanon. Results revealed that the Arabic version of the MDAS has good validity and reliability. Being female, having previous bad dental experiences during childhood and adolescence; reporting having dental fear and a sensation of nausea during dental treatment are risk factors for developing dental anxiety and phobia. Targeting these factors may improve the effectiveness of strategies to decrease anxiety and phobia among adult patients seeking dental care.

## Data Availability

Data are available from the corresponding authors upon reasonable request.

## Abbreviations

MDAS-A: Lebanese Arabic version of the Modified Dental Anxiety Scale
VAS: Visual analogue scale
SPSS: Statistical Package for Social Sciences
SD: Standard deviations
EFA: The exploratory factor analysis
CFA: Confirmatory factor analysis
χ^2^: Chi-square
df: Degrees of freedom
RMSEA: Root Mean Square Error of Approximation
GFI: Goodness of Fit Index
CFI: Comparative Fit Index
ROC: The receiver-operating curve
ICC: Intra-class correlation coefficient
KMO: Kaiser–Meyer–Olkin Test
EFA: Exploratory factor analysis
AUC: Area under the curve
OR: Odds ratio
CI: Confidence interval
